# Vaccination with recombinant paramyosin against the bovine lungworm *Dictyocaulus viviparus* considerably reduces worm burden and larvae shedding

**DOI:** 10.1186/s13071-015-0733-5

**Published:** 2015-02-24

**Authors:** Christina Strube, Claas Haake, Heinz Sager, Sandra Schorderet Weber, Ronald Kaminsky, Sandra Buschbaum, Deborah Joekel, Sabine Schicht, Elisabeth Kremmer, Julia Korrell, Thomas Schnieder, Georg von Samson-Himmelstjerna

**Affiliations:** Institute for Parasitology, University of Veterinary Medicine Hannover, Hanover, Germany; Novartis Centre de Recherche Santé Animale, St. Aubin, Switzerland; Helmholtz Zentrum München, German Research Center for Environmental Health, Institute of Molecular Immunology, Munich, Germany; Institute for Parasitology and Tropical Veterinary Medicine, Freie Universität Berlin, Berlin, Germany

**Keywords:** *Dictyocaulus viviparus*, Bovine lungworm, Parasitic bronchitis, Recombinant vaccine, Vaccination, Immunization, Paramyosin, Hidden antigen

## Abstract

**Background:**

The lungworm *Dictyocaulus viviparus*, causing parasitic bronchitis in cattle, induces a temporary protective immunity that prevents clinical disease. A radiation-attenuated larvae based vaccine is commercially available in a few European countries, but has the disadvantages of a live vaccine. As a recombinant subunit vaccine would overcome these disadvantages, the parasite’s muscle protein paramyosin (PMY) was tested as a recombinant vaccine antigen.

**Methods:**

*D. viviparus*-PMY was recombinantly expressed in *Escherichia coli* as a glutathione-S-transferase (GST)-fused protein. Emulsified in adjuvant Saponin Quil A, the protein was given intramuscularly into calves. Two independent recombinant PMY (rPMY) vaccination trials with negative control groups (first trial: adjuvant only; second trial: non-fused GST) as well as an additional positive control group in the second trial, using the Bovilis^©^Dictol live vaccine to verify vaccination results, were performed. To determine the vaccination success, shedding of larvae as well as worm burden and worm sizes were analyzed. Additionally, ELISA-based determination of development of immunglobulins IgM, IgA, IgE, IgG as well as the subclasses IgG1 and IgG2 was performed. To analyze PMY localization in the bovine lungworm, immunohistochemical staining of adult worms was carried out.

**Results:**

Immunohistochemical staining revealed that PMY is part of the bovine lungworm’s pharyngeal and body wall muscles. Vaccination with rPMY resulted in 47% [geometric mean: 67%] and 57% (geometric mean: 71%) reduction of larvae shedding in the first and second vaccination trial, respectively. Worm burden was reduced by 54% (geometric mean: 86%) and 31% (geometric mean: 68%), respectively, and worms of rPMY-vaccinated cattle were significantly shorter in both trials. Furthermore, ELISAs showed a clear antibody response towards rPMY with exception of IgE for which titers could not be detected. After challenge infection, rPMY antibodies were only exceptionally elevated among study animals indicating PMY to be a hidden antigen.

**Conclusions:**

Even though vaccination with the attenuated live vaccine was with 94% (geometric mean: 95%) reduction in larvae shedding and 93% (geometric mean: 94%) reduction in worm burden superior to rPMY vaccination, results using the latter are promising and show the potential for further development of a recombinant PMY-based vaccine against the bovine lungworm.

## Background

Dictyocaulosis in cattle, also known as parasitic bronchitis, is caused by the bovine lungworm *Dictyocaulus viviparus*. Cattle surviving the infection develop protective immunity lasting for up to one year. Based on the principle of induced protective immunological reactions, a live vaccine composed of irradiated third stage larvae (L3) was developed in the sixties of the last century by Jarrett et al. [1]. However, due to its high costs, limited shelf life and for ethical reasons, acceptance among veterinarians and farmers decreases nowadays. Therefore, control of the parasite relies primarily on anthelmintic treatment and pasture management.

The present study aimed to evaluate the potential of the antigen paramyosin as a recombinant subunit vaccine against the bovine lungworm in clinical vaccine trials. The muscle protein paramyosin (PMY) was firstly identified as a structural component of invertebrate muscle cells and is currently considered to be a significant part of the body wall and pharyngeal muscles of helminths [2-4]. PMY is usually associated with myosin and the core proteins and forms the macromolecular thick filaments [5,6]. Generally, its molecular weight is about 100 kDa and it is characterized by a two-chain alpha-helical coiled-coil structure [7]. Studies on various platyhelminths showed that PMY has the ability to bind collagen and interact with parts of the complement system, assumedly by binding to the collagen-like stalk regions of the complement subcomponent C1q [8,9]. Additionally, *Taenia crassiceps*-PMY was awarded the ability to bind the Fc part of antibodies [10]. Thus, a secreted or non-filamentous and surface-bound form of PMY may mask the worm against the host immune system [11]. Regarding nematodes, Zhang et al. [12] found that the outer membrane form of *Trichinella spiralis*-PMY plays an important role in the evasion of the host complement attack: Recombinantly expressed *T. spiralis*-PMY strongly bound to complement components C8 and C9 and inhibited the polymerization of C9 during the formation of the membrane attack complex. Strube et al. [13] demonstrated that recombinant PMY (rPMY) of *D. viviparus* was able to bind bovine IgG as well as bovine collagen. Thus, bovine lungworm-PMY might also have immunomodulatory functions protecting against host defense mechanisms, making it a potential vaccine target to control parasitic bronchitis. This assumption is substantiated by promising rPMY vaccination trials against trematodes like *Schistosoma* spp. and *Fasciola gigantica* [14-16]. In these studies, performed in the murine model and rabbits, respectively, rPMY vaccination led to an increase of specific antibody-titers in serum samples of vaccinated animals. Vaccination trials in large animal hosts like cattle, sheep or pigs with recombinant *S. japonicum*-PMY resulted in a partial protection against challenge infections: After immunization of pigs, 32-35% reduction of worm burden was observed [17]. When using the 3’ fragment of *S. japonicum*-PMY in the ovine host [18], 37% and 42% reduction of egg counts and 30% reduction of worms was reached, whereas native PMY obtained higher protection with 63% reduction of egg counts and 48% reduction of worms. McManus et al. [19] used full length rPMY of *S. japonicum* in combination with the adjuvant Quil A to vaccinate water buffaloes and achieved 49% reduction of egg counts and 34% reduction of worm burden. In nematodes, immunization with plasmid DNA encoding the *Brugia malayi*-PMY (BM5) induced development of strong antibody responses in mice [20,21]; however, vaccination of jirds did not result in reduced worm burdens [20]. Vaccination of BALB/c mice with recombinantly expressed *T. spiralis*-PMY achieved 36.2% reduction in muscle larvae burden following challenge infection [22]. The above results suggest PMY as a valuable helminth vaccine candidate why its protective potential against the bovine lungworm *D. viviparus* was evaluated in the present study.

## Methods

### Antigen preparation

*D. viviparus*-PMY was recombinantly expressed in One Shot® BL21 Star™ (DE3) chemically competent *E. coli* (Life Technologies, Germany) as a glutathione-S-tranferase (GST)-fused protein using the expression vector pET-41a (Merck Millipore, Germany). After purification as previously described by Strube et al. [13], rPMY was stored at −80°C in sterile 50 mM Tris buffer (pH 8.0) until use.

### Animals and immunization

In a first vaccination trial, ten parasite-naïve, male Holstein-Friesian calves, six to seven months of age (body weight 124 to 178 kg) were randomized into a vaccine group consisting of four cattle and a control group (adjuvant only-control) consisting of 6 cattle. In a second vaccination trial, 18 parasite-naïve, five to seven months old calves [Holstein-Friesian breed except one Simmental breed (Animal-ID: 553); bodyweight 139 to 182 kg] were randomized into a vaccine group, and two control groups [solely vaccinated with the vector-GST-tag (GST-control) or the commercially available live vaccine (Dictol group), respectively] of six animals each. Animal experiments were permitted by the ethics commission of the Lower Saxony State Office for Consumer Protection and Food Safety under reference number AZ509.6-42502-04/853. The second vaccination study was run under permit number D25A_05 of the Veterinary Authorities of the Canton of Fribourg in Switzerland.

In both vaccination trials, following an acclimatization period of seven days, cattle were immunized intramuscularly in the neck on study day 0, 21 and 42. The vaccine group of the first trial received 100 μg rPMY emulsified in 750 μg Quil A (Gerbu Biotechnik, Germany) as adjuvant (rPMY group) at each vaccination. The adjuvant only-control group received the same amount of adjuvant mixed with 50 mM Tris buffer (pH 8.0) instead of the antigen. The vaccine group of the second trial received again 100 μg rPMY emulsified in 750 μg Quil A. As rPMY contained a GST-tag at the N-terminus which might influence immunological reactions of the rPMY-vaccinated cattle, a GST-control group was included in the second trial. These calves received 100 μg “GST-tag” (*Schistosoma japonicum-*GST), which was produced by expression of the empty pET-41a expression vector. Again, Quil A was used as adjuvant. The Dictol group, which served as a positive control, was vaccinated with the Bovilis^©^Dictol vaccine (Bovilis® Huskvac, MSD Animal Health, Ireland). Accordingly, cattle were orally vaccinated on study day 14 and 42 with 25 ml larval suspension, each containing 1000–2000 viable *D. viviparus* third stage irradiated larvae. All animals were weighed prior to first immunization as well as necropsy to determine whether vaccination had an influence on weight gain.

### Challenge infection

On study days 62, 63, and 64 cattle were challenged by oral application with each 1100 infective third stage larvae (total of 3300 L3) of the *D. viviparus* isolate HannoverDv2000 in the first trial and with each 1000 L3 (total of 3000 L3) in the second trial. The *D. viviparus* isolate was maintained at the Institute for Parasitology, University of Veterinary Medicine Hannover, Germany.

### Determination of antibody development

During the first vaccine trial blood samples were taken weekly beginning from study day 0 until necropsy on study days 98/99. During the second trial, blood samples were available from day 0, 21, 42, 63 and 96 except the Dictol vaccine group, which was sampled on study day 14 (first Dictol vaccination) instead of study days 0 and 21. Obtained serum samples were stored at −20°C until analysis.

Antibody development against vaccinated rPMY was determined by ELISAs analyzing immunoglobulin classes IgM, IgA, IgE, whole IgG as well as the subclasses IgG1 and IgG2.

Nunc Amino Immobilizer 96 well plates (Thermo Scientific, Germany) were coated with 100 μl/well of 2.5 μg/ml rPMY dissolved in PBS by overnight incubation at 4°C. Plates were washed three times using PBS containing 0.05% Tween (PBST) before 100 μl of sera (diluted 1:40 in PBS in the first vaccination trial and 1:10 in the second trial) in LowCross Buffer® (CANDOR Bioscience, Germany; always diluted 1:2 with distilled water) were added to each well. For detection of IgE, sera were used undiluted. After one hour incubation at 37°C the wells were washed three times with PBST. Subsequently, 100 μl LowCross Buffer® containing 10 ng (first vaccination trial) or 20 ng (second vaccination trial) secondary antibodies was added to each well and incubated for 1 hour at 37°C. Secondary antibodies were sheep anti-bovine IgA:horseradish peroxidase (HRP), sheep anti-bovine IgG:HRP, sheep anti-bovine IgG1:HRP, sheep anti-bovine IgG2:HRP and sheep anti-bovine IgM:HRP (AbD Serotec, Germany). For IgE-detection, a monoclonal rat anti-bovine IgE antibody (5A2 IgG2a tissue culture supernatant), directed against the recombinantly expressed ε heavy chain constant region of bovine IgE was developed, and used at a 1:40 dilution in LowCross Buffer®. After 1 hour incubation at 37°C and three times washing with PBST, 100 μl/well LowCross Buffer® containing 20 ng HRP-conjugated mouse F(ab’)2 anti-rat IgG (H + L) antibody (1 mg/ml; Jackson ImmunoResearch Laboratories, USA) was added. Again, incubation conditions were 37°C for 1 hour. After removal of the final antibody solution, the wells were washed three times with PBST and wells were filled with 50 μl *o*-phenylenediamine hydrochloride (Sigma-Aldrich, Germany) dissolved in 25 mM citrate/50 mM phosphate buffer containing 0.04% hydrogen peroxide. After ten minutes of incubation at room temperature in the dark, the reaction was stopped by addition of 50 μl/well 2.5 M sulfuric acid. Optical density (OD) was measured at a wavelength of 490 nm using the ELx800™ Absorbance Microplate Reader (BioTek, Germany). All serum samples were analyzed as duplicates. For each set of duplicates, the mean was calculated for data analysis.

### Determination of shedding of larvae

Individual fecal samples were collected daily from study day 84 to 97 during the first vaccination trial and on study day 85, 89 and 92 during the second vaccination trial. An amount of 20 g (first vaccination trial) and 60 g feces (second vaccination trial) were processed using the Baerman technique followed by calculation of larvae number per 1 g feces (LPG). LPG values of each animal were summed and the overall arithmetic and geometric means were calculated. For geometric mean calculation, a logarithmic transformation (ln[count + 1]) was applied to individual LPG values to address skewness of the data, as well as zero counts. Back-transformed geometric means were calculated by e^x^ − 1, where x equals the mean of logarithmically transformed values. Arithmetic and geometric standard deviations (SD) were calculated. Arithmetic SD (SD_a_) was calculated as the square root of variance, whereas geometric SD (SD_g_) was calculated as follows:

$$ {\mathrm{SD}}_{\mathrm{g}}= exp\left(\sqrt{\frac{1}{n}{\displaystyle \sum_{i=1}^n}{\left[ ln\left(\frac{x_i}{{\overline{x}}_g}\right)\right]}^2}\right) $$

In contrast to SD_a_, SD_g_ is a multiplicative factor and therefore dimensionless.

Reduction in larvae shedding based on arithmetic or geometric means was calculated as follows:

$$ \mathrm{Reduction}\ \left[\%\right]=\left(1-\mathrm{Mean}\ \mathrm{larvae}\ \mathrm{counts}\ \mathrm{vaccinated}\ \mathrm{group}/\mathrm{Mean}\ \mathrm{larvae}\ \mathrm{counts}\ \mathrm{control}\ \mathrm{group}\right) \times 100 $$

Statistical analyses were done using *t*-test or Mann–Whitney-Rank sum test if the data failed the normality test (SigmaStat software, version 3.1, Systat Software, Germany). A p-value ≤0.05 was considered statistically significant.

### Determination of worm burden and worm size

After necropsy on study days 98 and 99 (first trial) as well as study days 96, 97 and 98 (second trial), lungs of individual cattle were removed. To retrieve adult worms, lungs were carefully dissected; the tracheoles, bronchi and bronchioles were opened with scissors and all visible parasites were taken out using tweezers. In the second vaccination trial, lungs were additionally perfused via the pulmonary artery. Worms were counted and additionally separated by sexes in the first trial. In case of damaged worms, only tails were counted and sexed. Reductions in worm burden based on arithmetic and geometric means as well as statistical analyses were calculated as described above.

To estimate if the vaccine has an influence on adult worm size, 25 male and 25 female worms per animal were investigated by lengths and widths measurements. If less than 25 worms per sex and cattle were collected, all available worms per sex were measured. For this purpose, worms were plated on a black pad and photographed. For subsequent size measurements cell^B^ Basic imaging software (version 3.1, Olympus Soft Imaging Solutions, Germany) was used. The overall group mean worm length or width was calculated on basis of all individual worms belonging to the respective vaccination group. Statistical analyses were performed as described above.

### Immunohistochemical staining

Cryosections of adult *D. viviparus* in Leica Jung Tissue freezing medium (Leica, Germany) were prepared using the Cryotome Leica 2800E Frigocut Microtome Cryostat (Leica, Germany). Longitudinal- and cross-cryosections (10 μm thick) were produced, transferred to Superfrost™ plus slides (Thermo Scientific, Germany) and dried in a desiccator for 48 hours. Ice cold (−20°C) acetone was added for fixation [10 minutes at room temperature (RT)] and slices were subsequently dried at RT and afterwards rinsed with PBS. After carefully dabbing and encircling the tissue with a DAKO-pen, a monoclonal anti-*C. elegans*-PMY antibody (1.85 μg/ml; available under the designation 5–23 from the Developmental Studies Hybridoma Bank, USA) diluted 1:10 in PBS-5% goat serum was dripped on the slides followed by 1 hour incubation at RT. Slides were then rinsed two times with PBS and once with double distilled water. Goat anti-mouse IgG-FITC (1 mg/ml; AbD Serotec, Germany) was used as secondary antibody in diluted 1:1000 in PBS (final concentration 5 μg/ml). Incubation was again 1 hour at RT in the dark, followed by two times washing with PBS and once with double distilled water before drying at RT. Afterwards, slices were covered with 100 μl Mowiol including 1:200 diluted DAPI (1 μg/ml, Sigma-Aldrich, Germany) as mounting medium and allowed to dehydrate for approximately 24 hours in the dark. Sections were visualized with a fluorescence microscope (Nikon Eclipse Ti, Nikon, Germany) and photographed using the software NIS-elements AR 64 bit (version 2.3, NIKON, Düsseldorf, Germany).

## Results

### Conditions of the calves

In the second trial, three animals (one of the GST-control group, one of the rPMY group and one of the Dictol vaccine group) had to be euthanized before challenge infection due to severe weight loss and diarrhea independently from treatment. Therefore, results include five cattle per group only. Three animals of the GST-control group (animal ID 412, 361 and 553) had to be treated with antibiotics (Marbocyl^©^, Vétoquinol, WDT, Germany) for up to 5 days and the remaining two animals of the group had also to be treated with anti-inflammatory meloxicam (Metacam^©^, Boehringer Ingelheim Vetmedica, Germany) for 2 days. The Simmental calf (animal ID 553) did not recover and had to be euthanized three days before the initially planned necropsy due to severe dictyocaulosis, but was subjected to parasitological necropsy and results were included in the study. Remaining cattle of the trials showed spontaneous coughing and intensified respiratory sounds occasionally.

In the second trial, the average weight gain of cattle (Table 1) belonging to the rPMY and Dictol group were considerably higher than those of the GST-control group; however, results were not statistically significant.

**Table 1:** **Vaccination parameters weight gain, larvae counts and worm burden**

**Animal ID**	**Weight gain [kg]**	**Larvae counts [LPG]** ^*^	**Total worm count**	**Male worm count**	**Female worm count**
**Trial 1**					
**Adjuvant only-control group**					
110	59	1047	1136	447	689
795	58	569	798	324	474
802	62	53	213	77	136
805	60	302	453	159	294
808	66	304	433	142	291
814	59	153	651	260	391
Arithmetic mean	61.7	404.7	614.0	234.8	379.2
SD_a_	2.9	359.8	324.7	136.3	189.4
Geometric mean	60.6	276.7	539.7	200.9	337.9
SD_g_^**^	1.0	2.8	1.7	1.8	1.7
**rPMY group**					
806	69	609	925	351	574
812	49	131	4	1	3
813	76	7	64	21	43
822	70	116	126	60	66
Arithmetic mean	66.0	215.8	279.8	108.3	171.5
SD_a_	11.7	267.9	433.0	163.7	269.6
Geometric mean	65.1	92.2	77.6	30.2	50.0
SD_g_^**^	1.2	5.0	7.0	8.5	6.5
Reduction [%]^***^		46.7 (66.7)	54.4 (85.6)	53.9 (85.0)	54.8 (85.2)
**Trial 2**					
**GST-control group**					
361	22	67	940		
129	n.a.	12	42		
412	30	128	1101		
269	53	84	873		
553	18	201	1871		
Arithmetic mean	30.8	98.4	965.4		
SD_a_	15.6	70.8	652.0		
Geometric mean	28.2	71.2	591.4		
SD_g_^**^	1.5	2.6	3.8		
**rPMY group**					
699	39	87	1019		
485	56	73	1192		
034	70	25	917		
906	76	0	0		
887	23	26	209		
Arithmetic mean	52.8	42.2	667.4		
SD_a_	21.9	36.4	528.4		
Geometric mean	48.6	20.5	187.0		
SD_g_^**^	1.1	n.a.	n.a.		
Reduction [%]^***^		57.1 (71.2)	30.9 (68.4)		
**Dictol vaccine group**					
214	48	1	2		
688	36	13	152		
598	79	1	63		
221	48	1	50		
825	46	16	56		
Arithmetic mean	51.4	6.4^§^	64.6		
SD_a_	16.2	7.5	54.4		
Geometric mean	49.7	3.5	37.6		
SD_g_^**^	1.1	1.5	1.6		
Reduction [%]^***^		93.5 (95.0)	93.3 (93.6)		

SD_a_ = arithmetic standard deviation, SD_g_ = geometric standard deviation, n.a. = not available.

^*^Values represent the sum of fecal larvae counts from day 84 to 97.

^**^SD_g_ is a multiplicative factor and therefore dimensionless.

^***^Percent reduction based on arithmetic mean with values in brackets representing percent reduction based on geometric mean.

^§^p ≤ 0.05.

### Parasitological parameters (larvae shedding, worm burden and worm size)

Reduction of larvae shedding in rPMY-vaccinated cattle based on arithmetic mean was 46.7% (geo. mean: 66.7%) in the first and 57.1% (geo. mean: 71.2%) in the second vaccination trial. Arithmetic mean LPG of the Dictol vaccine group was with 93.5% reduction of larvae shedding statistically significantly lower compared to the GST-control group. Adult worm burden was reduced by 54.4% (geo. mean: 85.6%) in rPMY-vaccinated animals of the first trial and 30.9% (geo. mean: 68.4%) in the second trial. Reduction of worm burden of the Dictol vaccine group was 93.3% (geo. mean: 93.6%). Detailed data are listed in Table 1.

Adult lungworm measurements of the rPMY-vaccinated cattle of the first trial showed significantly reduced worm lengths, while widths were significantly enlarged. In the second trial, worm lengths as well as widths of the rPMY-vaccinated group were significantly smaller than those of the GST-control group. The average length and width of female worms of the Dictol vaccine group were significantly smaller than those of the GST-control. This was also true for the average width but not length of male worms. Results of lungworm size measurements are summarized in Table 2.

**Table 2:** **Worm size measurements**

	**No. of worms counted**	**Male worms**		**No. of worms counted**	**Female worms**	
**Animal-ID**		**Mean length [mm]**	**Mean width [mm]**		**Mean length [mm]**	**Mean width [mm]**
**Trial 1**						
**Adjuvant only-control group**						
110	25	40.32	0.43	25	55.47	0.47
795	25	36.71	0.41	25	47.17	0.43
802	25	29.90	0.30	25	40.56	0.34
805	25	34.02	0.37	25	47.99	0.44
808	25	35.38	0.38	25	46.07	0.41
814	25	35.39	0.35	25	46.46	0.41
Sum/overall mean	150	35.29	0.37	150	47.29	0.42
SD		4.64	0.06		6.59	0.06
**rPMY group**						
806	25	38.21	0.49	25	51.08	0.52
812	1	29.97	0.43	3	40.55	0.48
813	12	29.45	0.39	18	36.65	0.43
822	25	31.72	0.46	24	41.57	0.46
Sum/overall mean	113	33.84*	0.46*	70	43.66*	0.49*
SD		4.93	0.08		7.64	0.08
**Trial 2**						
**GST-control group**						
361	25	31.71	0.33	25	39.89	0.40
129	11	26.03	0.28	25	32.63	0.34
412	25	32.95	0.35	25	49.25	0.45
269	25	34.82	0.36	25	46.04	0.40
553	25	32.74	0.35	25	44.30	0.40
Sum/overall mean	111	32.35	0.34	125	42.42	0.40
SD		3.91	0.05		7.11	0.07
**rPMY group**						
699	24	28.55	0.27	25	38.37	0.28
485	25	33.23	0.30	25	40.56	0.36
034	25	29.66	0.29	25	39.88	0.37
906	0	-	-	0	-	-
887	25	28.32	0.33	25	35.30	0.37
Sum/overall mean	99	29.95*	0.30*	100	38.53*	0.34*
SD		3.11	0.05		4.44	0.06
**Dictol vaccine group**						
214	0	-	-	2	23.22	0.36
688	14	35.93	0.28	25	40.14	0.39
598	1	31.18	0.27	2	36.18	0.39
221	11	24.52	0.30	19	29.96	0.31
825	2	26.13	0.28	20	30.68	0.31
Sum/overall mean	28	30.58	0.29*	48	33.90*	0.34*
SD		6.59	0.03		6.45	0.07

SD = standard deviation.

*p ≤ 0.05.

### Determination of antibody response to vaccination

All rPMY-vaccinated animals developed antibodies in response to vaccination. Most prominent immunoglobulin (sub)classes were IgG including IgG1 and IgA, whereas no IgE response was detectable in the serum samples. The latter also applies to all tested serum samples with exception of one GST-control group animal (ID 412) at study day 98, at which a weak IgE increase was observed.

The GST-control animals of the second trial (but not the adjuvant only-control of the first trial) developed antibodies in response to vaccination as well, but with less intensity compared to rPMY-vaccinated animals. Again, most prominent immunoglobulin (sub)classes were IgG including IgG1.

**Figure 1:**
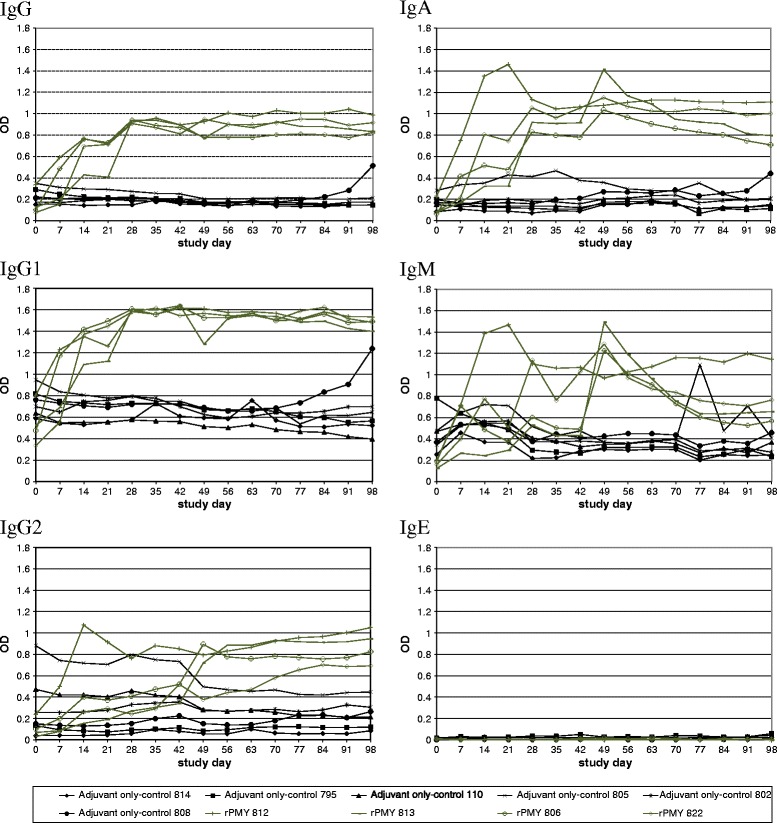
**Anti-rPMY-antibody development during the first vaccination trial.** Optical density (OD) values over time are for immunoglobulin (Ig) classes IgG, IgG1, IgG2, IgM, IgA, IgM and IgE for the individual study animals. The graph legend lists animal IDs of each group.

**Figure 2:**
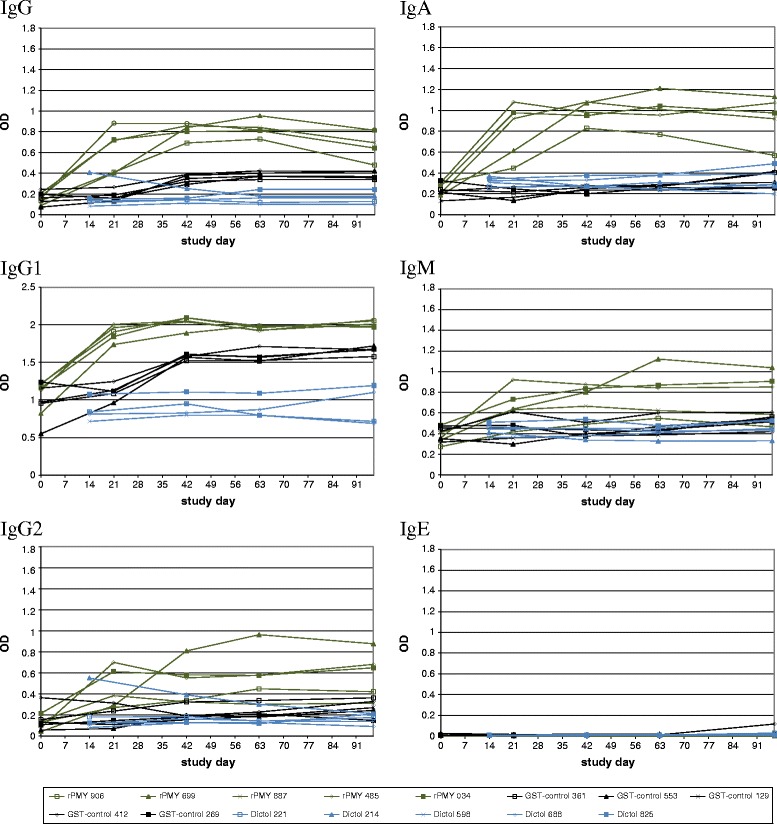
**Anti-rPMY-antibody development during the second vaccination trial.** Optical density (OD) values over time are for immunoglobulin (Ig) classes IgG, IgG1, IgG2, IgM, IgA, IgM and IgE for the individual study animals. The graph legend lists animal IDs of each group.

After challenge infection, no increase in antibody intensity was detected within any of the groups, except of animal no. 808 (adjuvant only-control, trial 1), which showed a distinct IgG and IgG1 increase and a slight IgA increase. Furthermore, animal no. 805 (adjuvant only-control, trial 1) showed increased IgM levels. Sera of the Dictol group did not show any antibody response to rPMY. Determined anti-PMY-antibody curves are shown in Figure 1 (first vaccination trial) and Figure 2 (second vaccination trial).

### Immunohistochemical staining

**Figure 3:**
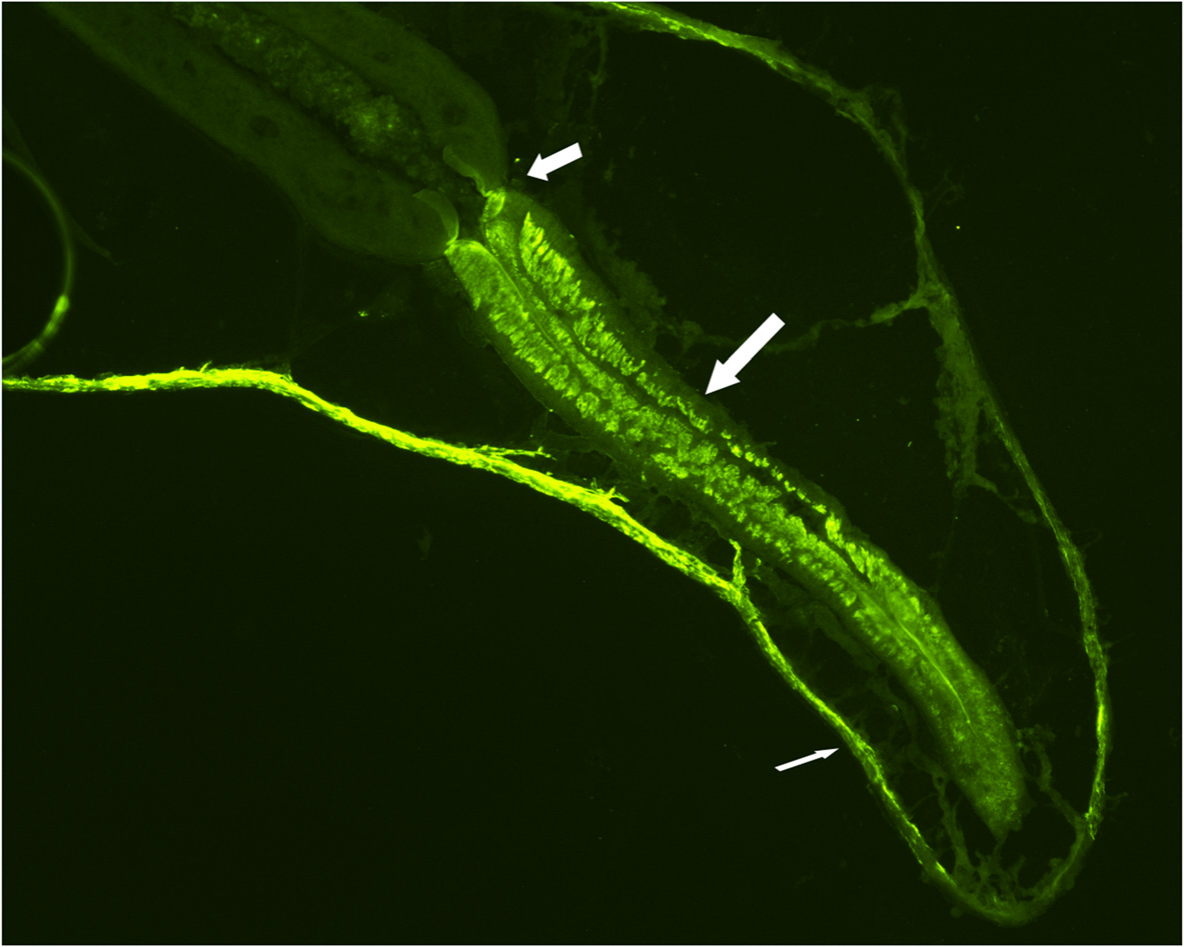
**Localization of PMY in** ***D. viviparus.*** Immunohistochemical staining localized PMY in the pharyngeal and body wall muscles of the bovine lungworm. The long thick arrow indicates the pharynx, the short thick arrow the transition into the oesophagus. The thin arrow indicates the cuticle and underlying body wall muscles.

FITC-labeled antibodies directed against anti-*C. elegans*-PMY antibodies reacted strongly with the pharyngeal muscles and longitudinal somatic muscles of the body wall (Figure 3). No staining was seen in the negative control sections.

## Discussion

The development of a recombinant subunit vaccine against a helminth parasite would be a milestone in parasite control of livestock. This study intended to test the protective potential of bovine lungworm-rPMY as a vaccine candidate and in both conducted trials, vaccination with rPMY resulted with 54.4% (geo. mean: 85.6%) and 30.9% (geo. mean: 68.4%) in a distinct reduction of worm burden, showing the protective capacity of rPMY. Several attempts have been made to identify possible candidate proteins for a recombinant subunit vaccine against *D. viviparus* [23-26], but to date only one in vivo study in cattle has been published using acetylcholinesterase as recombinantly *E. coli*-expressed His-tagged protein combined with Incomplete Freund’s adjuvant [27]. However, in contrast to the presented rPMY trials, acetylcholinesterase did not lead to a reduced worm burden or larval shedding.

Compared to other trials with recombinant proteins as vaccine candidates against cattle nematodes, e.g. the polyprotein allergen (OPA) [28] or activation-associated secreted proteins (ASPs) [29] of *Ostertagia ostertagi*, the present study exhibits the highest level of protection of a single recombinant antigen against a cattle nematode species so far. In sheep, vaccination against *Haemonchus contortus* resulted in a protection up to 95% regarding worm burden and up to 99% regarding egg shedding by use of native proteins like the gut membrane protein H11 and glycoprotein complex H-gal-GP as vaccine candidates [30-33]. However, using the recombinant form of H11 as antigen, only 30% reduced worm burden was observed [34]. This and other studies [28,29] indicate that results achieved for isolated native proteins cannot be extrapolated to recombinant proteins and results are not readily comparable to those obtained with recombinant proteins.

The lower percentage reduction in worm burden of rPMY-vaccinated cattle in the second trial might be explained by a change in the control group: While in the first trial the control was vaccinated with the adjuvant in Tris buffer only (adjuvant only-control group), the control animals (GST-control group) of the second trial received additionally recombinant GST fusion protein expressed from the expression vector without PMY-insert (“empty vector”). GST is known to induce an antibody response in experimental animals [35] and moreover can induce a partial protection against parasitic worms, including trematodes [36] as well as nematodes [37,38]. Hence, GST-tagged proteins may increase the vaccination success, which is why the GST-tag has not been removed from rPMY prior to vaccination. It might be speculated that the antibodies formed against the vector’s *S. japonicum*-GST cross reacted with *D. viviparus*-GST - with the consequence that the differences between the GST-control and rPMY group became smaller in the second trial.

In addition to reductions in worm counts, it was observed that worms of rPMY-vaccinated groups were significantly shorter compared to those of the control groups, independently of their sex. Since immunohistochemistry showed that PMY is part of the bovine lungworm body wall and pharyngeal muscles, it can be assumed that vaccination-induced antibody development had an influence on the parasite’s development. Similar observations were made in flies, in which antibodies directed against muscular elements passed through the gut and interfered with function and growth of the muscular system [39,40]. As early passive transfer studies using hyper-immune sera revealed that antibodies play a major role for the protective immunity against the bovine lungworm [41], antibody development against rPMY was analyzed. In both trials, rPMY-vaccinated animals showed immunological reactions against the vaccine antigen. The most distinct increases were observed for IgG and the subclass IgG1 as well as IgA. These immunoglobulins are secreted onto the bovine mucosa [42-44] and the observed reductions in parasitological parameters might at least partially be attributed to them. When comparing results of rPMY receiving animals with the respective control group (trial 1: adjuvant only-control group; trial 2: GST-control group), antibody titers of adjuvant only-control animals of the first trial remained more or less on the same level as at the beginning of the trial. By contrast, IgG as well as IgG1 and to a much lesser extend IgA titers of the GST-control group increased in response to vaccination. It has to be noted, however, that these ELISA results probably represent the reaction of developed anti-GST antibodies with the GST-part of the coated PMY fusion protein. The missing IgE-development against rPMY could either be explained by the general lack of free IgE in sera since it is bound to cell surfaces of e.g. basophilic granulocytes, or a general lack of a specific IgE response against rPMY. The lack of any Ig-increase in the Dictol-control group as well as the lacking increase in all but two study animals after challenge infection indicate that PMY is a hidden antigen. A counter-argument to this assumption is a possible unresponsiveness of antibodies developed against rPMY to native PMY. However, immunohistochemistry results which demonstrated the pharyngeal and longitudinal somatic muscles of the body wall as PMY location strongly support the hidden antigen hypothesis.

In the present study most results achieved were not statistically significant, due to the high variance of results, especially those for worm burden and larvae counts. This problem has already been described previously [41] and becomes obvious in the lacking statistical significance of 93.3% (or 93.6% when considering the geometric mean) reduction in worm burden regarding the Dictol vaccine group. A substantial increase of group sizes could solve the problem, but was not considered since the performed vaccination trials represented pilot studies.

A general challenge regarding vaccination with recombinant *D. viviparus*-PMY might be the occurrence of amino acid substitutions among three PMY transcript variants [13]. Vaccination trials against *F. hepatica* with recombinant fatty acid binding protein resulted in a reduced protection compared to the native protein fraction. The authors explained this outcome by the occurrence of eight different isoforms of the mentioned protein and that the one used for the recombinant vaccine was probably less immunogenic [45]. In addition, there is the possibility that naturally occurring isoforms are not recognized by antibodies formed against a recombinant isoform when the difference is part of the epitope. However, the observed 10 amino acids substitutions among the 876 deduced amino acids of bovine lungworm PMY [13] should not affect binding of the majority of developed antibodies among PMY variants.

General approaches for improvement of vaccination success could include a change of the expression system. Since *D. viviparus* is an eukaryotic organism, an expression system capable of post-translational modifications might deliver a rPMY which resembles the original more closely. Prediction of N-glycosylation sites for *D. viviparus*-PMY using the GlycoEP web server [46] resulted in a potential N-glycosylation at amino acid position 653 (data not shown). This could be of relevance since it has been suggested that *D. viviparus*-proteins carry immune dominant N-glycan moieties which influence the immune response during infection as well as after live vaccination [47]. Nevertheless, the ultimate goal of vaccinations against nematodes is not necessarily a complete prevention of the establishment of worms, but the prevention of health problems, avoidance of economic losses and at the same time the possibility for the development of a stable immunity by natural booster infections. According to Barnes et al. [48], a substantial vaccination benefit against gastrointestinal worm infection of sheep can be achieved already when the vaccine protects about 80% of the flock with an efficiency of 60%. Whether or not these values can also be applied for the bovine lungworm needs to be evaluated in appropriate studies including model calculations.

## Conclusions

The vaccine antigen rPMY partially protected cattle against *D. viviparus* infection, although the live vaccine Bovilis^©^Dictol induced a higher reduction in worm burden and shedding of larvae. Furthermore, rPMY revealed significant shorter worms after challenge infection besides reduction of worm burden and larvae shedding. In all, achieved reductions in worm burden, worm size and shedding of larvae make rPMY a promising candidate for further vaccination studies offering multiple possibilities for further improvements. Such improvements might be an eukaryotic expression system or the replacement of the adjuvant, since many different parameters of the immune response are influenced by the adjuvant and the vaccination success can be heavily influenced by their choice [49]. Furthermore, combinations of different recombinant *D. viviparus* proteins may also have an additive or synergistic effect.
